# Effects of naturopathic medicines on Multiplate and ROTEM: a prospective experimental pilot study in healthy volunteers

**DOI:** 10.1186/s12906-016-1051-y

**Published:** 2016-02-17

**Authors:** August Bagge, Ulf Schött, Thomas Kander

**Affiliations:** Medical Faculty, University of Lund, Box 117, 221 00 Lund, Sweden; Department of Clinical Sciences, University of Lund, Box 188, SE-221 00 Lund, Sweden; Department of Intensive and Perioperative Care, Skåne University Hospital Lund, 22185 Lund, Sweden

**Keywords:** Echinacea, Fish oil, Ginkgo biloba, Ginseng, St. John’s wort, Valeriana, Garlic, Platelet function, Coagulation

## Abstract

**Background:**

Of patients undergoing surgery, 22 to 57 % have been reported to be using naturopathic medicines. Several of these medicines have been reported to increase bleeding or enhance the effect of other drugs that increase bleeding. The Swedish Medical Products Agency recommends cessation of the use of the naturopathic medicines echinacea, fish oil, ginkgo biloba, ginseng, St. John’s wort, valeriana and garlic 2 weeks before surgery. The aim of this pilot study was to examine the effects of these 7 naturopathic medicines in healthy humans by utilising multiple electrode aggregometer (Multiplate) and viscoelastic rotational thromboelastometer (ROTEM) to obtain data for sample size calculation before a larger trial.

**Methods:**

Thirty-five healthy volunteers ingested one of the listed naturopathic medicines for 7 days. Each naturopathic medicine was taken in a recommended standard dose by 5 volunteers. ROTEM clot initiation (CT), clot formation (CFT), α-angle (AA) and clot structure (MCF) were analysed with tissue factor activated (EXTEM) and native (NATEM) assays. The Multiplate platelet aggregation area under curve (AUC) was measured with adenosine diphosphate (ADP), collagen (COL) and arachidonic acid (ASPI) assays.

**Results:**

Multiplate with ADP agonist decreased from 73 ± 8.7 AUC to 60 ± 5.9 AUC (*P* = 0.003, 95 % confidence interval (CI) −19.2 to −7.6) after medication with fish oil, but fish oil had no effect on COL or ASPI reagents. None of the other naturopathic medicines had any effect on Multiplate aggregometry. ROTEM NATEM-CFT increased from 217 ± 32 s to 283 ± 20 (*P* = 0.009, 95 % CI 26.8 to 107), and NATEM-AA decreased from 52 ± 3.9° to 44 ± 2.3° (*P* = 0.009, 95 % CI −12.0 to −3.2) after medication with fish oil. There were no significant changes in the other NATEM or EXTEM parameters. The other naturopathic medicines had no significant effects on ROTEM or Multiplate aggregometry.

**Conclusions:**

We have demonstrated that a recommended standard intake of 1260 mg Ω-3 polyunsaturated fatty acids (fish oil) daily – but not echinacea, ginkgo biloba, ginseng, St. John’s wort, valeriana or garlic – may decrease platelet aggregation and clot formation. A larger trial in this setting would be meaningful to perform.

**Trial registration:**

Trial registration ISRCTN78027929. Registered 19 May 2015.

## Background

Of patients undergoing surgery, 22 to 57 % have been reported to be using naturopathic medicines [1, 2]. Although evidence based data of increased perioperative bleeding are lacking [3] several naturopathic medicines may increase the risk of bleeding or enhance the effect of other drugs that may increase bleeding, such as non-steroid anti-inflammatory drugs [4]. The recommendation of the Swedish Medical Products Agency [5] is to stop the usage of the naturopathic medicines echinacea, fish oil, ginkgo biloba, ginseng, St. John’s wort, valeriana and garlic 2 weeks before elective surgery to avoid unwanted bleeding effects.

The mechanism for increased bleeding may include inhibitory effects of these medications on platelet functions mediated through several different pathophysiological pathways [6], including adenosine diphosphate- (ADP) [7], collagen- (COL) [8] and adrenaline-induced inhibition of platelet aggregation [9]. Furthermore, thromboxane B2 levels [10], platelet P-selectin expression [11], platelet CD63 expression [12] and arachidonic acid levels of platelet phospholipids [13, 14] may also be affected. In the past, measuring platelet function was a difficult and laborious process, but it has recently been made more accessible through different point-of-care (POC) techniques. Thromboelastography measures the dynamic interaction between aggregating platelets and polymerised fibrin and the final strength of a blood clot in whole blood, whereas routine plasma-based coagulation tests measure only the time until clotting begins [15]. There is observational evidence that 2 POC viscoelastic devices, thromboelastography (TEG) and rotational thromboelastometer (ROTEM), may be superior to routine tests in monitoring and guiding hemostatic resuscitation in bleeding patients [15]. However these techniques are not proven to reduce mortality [16] and several concerns have been raised using the viscoelastic devices bedside by non-laboratory personal because these tests are hard to standardize [17] and some authors describe their predictive performance not consistently superior to routine tests [18]. Furthermore, TEG and ROTEM cannot measure the effects of weak platelet-inhibiting drugs like acetylsalicylic acid (ASA) nor strong platelet-inhibiting drugs such as ADP-inhibitors. Therefore, different POC platelet aggregation tests use different platelet agonists to monitor the effects of ASA and ADP-inhibitors. One such test is multiple electrode aggregometry (MEA; Multiplate).

Neither ROTEM nor TEG has been used to monitor naturopathic medicines. Fish oil has been shown to reduce platelet collagen receptor function, as detected by the Multiplate COL [19] and ADP tests [11]. A review article mentions reduction of thromboxane A2 levels as a possible mechanism of garlic and fish oil [20]. ASA also inhibits thromboxane A2, and therefore Multiplate analysis with an arachidonic acid agonist designed to detect ASA platelet inhibition (Multiplate ASPI test) might have the potential to detect effects of naturopathic medicines.

The aim of this pilot study was to examine the effects in healthy volunteers of 7 days of peroral intake of echinacea, fish oil, ginkgo biloba, ginseng, St. John’s wort, valeriana and garlic naturopathic medicines (Table 1) on Multiplate aggregometry and ROTEM and to obtain data for sample size calculation before a larger trial.

**Table 1:** Standard dosages as described on the packages were used for 7 days

Naturopathic medicine	Grams of active substance/day
Echinacea	49.6 mg dried root and herb
Fish oil	1260 mg Ω-3 polyunsaturated fatty acids
Ginkgo biloba	200 mg extract (represents 3–4 g dried ginkgo leaf)
Ginseng	200 mg extract (represents 210 mg dried ginseng root)
St John’s wort	120–219 mg extract (represents 0,39–0,87 g dried herb)
Valeriana	400 mg extract (approximately 2,4 g dried root)
Garlic	1260 mg garlic oil macerate (represents 3 mg allicin)

## Methods

**Fig. 1:**
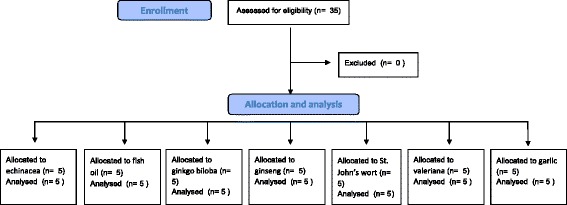
Flow chart

The study was approved by the Regional Ethical Review Board, Lund, Sweden (registration number 2010/482). Thirty-five non-smoking, healthy, Caucasian volunteers (16 men and 18 women, ages 21 to 62 [median 27 and mean 36 years old]) gave signed consent to ingest one of the listed naturopathic medicines for 7 days (Fig. 1). Volunteers were all staff members at the intensive care unit, University Hospital, Lund, Sweden, and were considered to have normal laboratory parameters. Exclusion criteria were pregnancy, planned surgery, or intake of any kind of anticoagulant or antithrombotic medicine, including recent intake of aspirin or non-steroid anti-inflammatory drugs. Five volunteers took each naturopathic drug (echinacea, fish oil, ginkgo biloba, ginseng, St. John’s wort, valeriana and garlic) in a recommended standard dose (Table 1). No differences in age or sex were found between the treatment groups.

The test period of 7 days was chosen with the normal 7- to 8-day lifespan of platelets in mind [21]. When assessing platelet function and coagulation after the test period, most platelets in circulation would have been exposed to the current medication. Recommended standard dosing was selected because we wanted to test the effect of naturopathic medicines in a clinically relevant and common dose.

### Blood sampling

Venous blood was collected using the venipuncture technique (Vacutainer) before and after 7 days of naturopathic medication. First, 3 ml of blood was discarded. For the ROTEM analysis, a 2.7 mL tube containing 0.109 M citrate (3.2 % citrate, BD Vacutainer systems, Plymouth, UK) was used, and for the Multiplate analysis, blood was collected in a 3.0 mL Hirudin tube (Dynabyte GmbH, Munich, Germany).

### Viscoelastic coagulation analysis

The viscoelastic coagulation instrument ROTEM (Pentapharm, Munich, Germany) was chosen to evaluate if the platelet contribution to secondary haemostasis is affected after ingestion of the selected naturopathic medicines. Blood was stored at 37 °C according to manufacturer instructions, and ROTEM tests were performed within 120 min after sampling. ROTEM is a viscoelastic test that measures coagulation in whole blood. Whole blood (300 μL) is installed in a fixed disposable cuvette with a rotating pin. When the blood starts to clot, a fibrin bridge is created between the pin and the cup, and the resistance to rotation is displayed as a graph that indicates the time for the blood clot to form and the strength of the clot. For all ROTEM analyses, 20 μL of 0.2 M CaCl2 (StartTem) was added to block the citrate anticoagulant. Three different ROTEM assays were used. EXTEM and FIBTEM are tissue factor activated and NATEM is conducted without coagulation activating reagents. The EXTEM assay is influenced by extrinsic coagulation factors, platelets and fibrinogen, whereas the FIBTEM assay provides information regarding the functional fibrinogen concentration and fibrin stability of the clot.

Parameters for the EXTEM and NATEM assays were clotting time (CT), clot formation time (CFT), α-angle (AA) and maximum clot firmness (MCF). CT represents the time from adding the start reagent until the clot starts to form. CFT gives information about the initial speed of the formation of the clot, AA displays the rate at which a solid clot forms, and MCF indicates the maximal strength of the clot. Together the parameters provide an overview of the coagulation process. Only MCF was recorded for the FIBTEM analyses.

### Impedance aggregometry

Platelet aggregation was measured using Multiplate (Roche Diagnostics Scandinavia AB, Bromma, Sweden). Tests were performed between 30 min and 2 h after blood sampling. Multiplate measures platelet aggregation by impedance aggregometry. The device has 5 channels by which platelet aggregation after the addition of different platelet agonists can be simultaneously measured. The test cells contain 2 independent silver-coated copper wires between which electricity is conducted. As the platelets aggregate on the wires, the electrical impedance in the electrical circuit is increased and presented as a graph where the area under curve (AUC) quantifies the aggregation. In this study, 3 different platelet agonists were used: adenosine diphosphate (ADP) agonist (6.5 μM), collagen (COL) agonist (3.2 μg/ml) and arachidonic acid (ASPI) agonist (0.5 mM).

### Statistical analysis

All variables were treated as normally distributed and are presented as mean ± standard deviation (SD). Paired t-tests were used to test for statistically significant differences before and after administration of the naturopathic medicines. To reduce the risk of a Type I error due to multiple testing, the significance level was set at a *P*-value of <0.01. All statistical analyses were performed using GraphPad Prism 6 (GraphPad Software, Inc., La Jolla, CA, USA).

## Results

### Multiplate

**Fig. 2:**
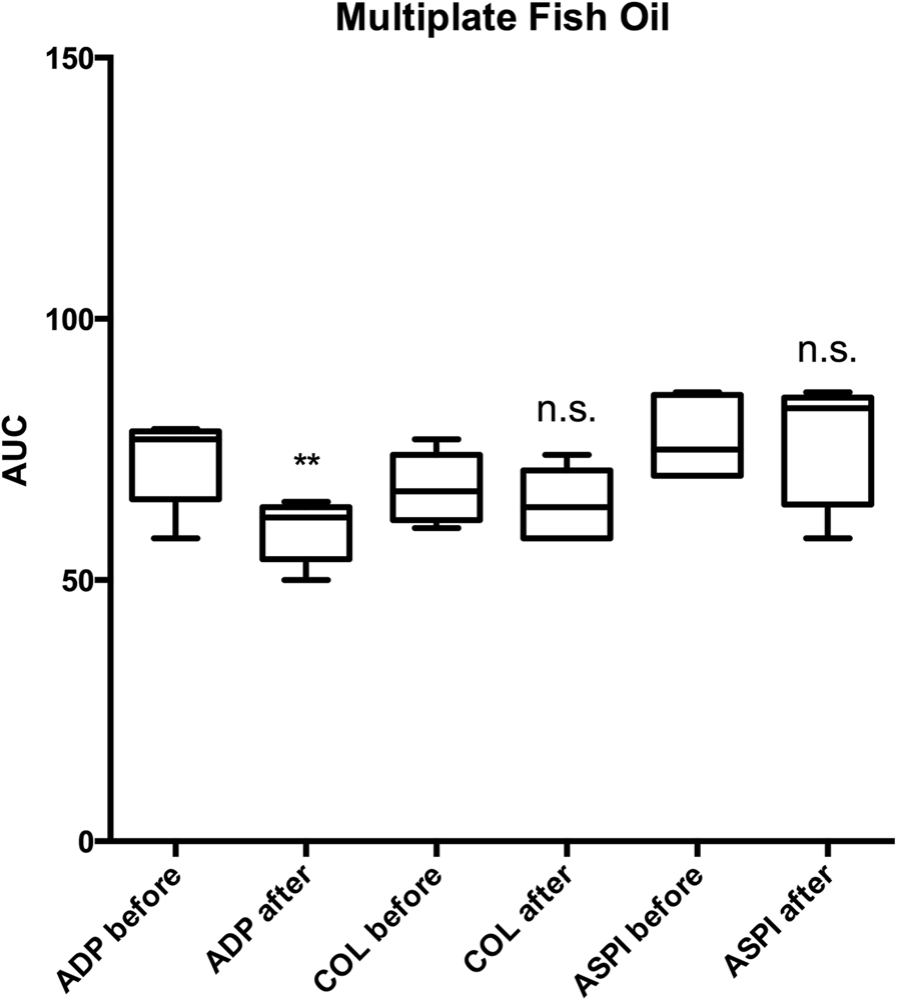
Results from Multiplate analysis of whole blood before and after 7 days of fish oil medication. Before and after results are compared using paired *t*-test. **P < 0.01. n.s. = non-significant

Multiplate with ADP agonist decreased from 73 ± 8.7 AUC to 60 ± 5.9 AUC (*P* = 0.003, 95 % confidence interval (CI) −19.2 to −7.6) after 7 days of fish oil intake (Fig. 2 and Table 2). COL and ASPI did not show this effect. None of the other naturopathic medicines had any significant effects on Multiplate aggregometry.

**Table 2:** Results from Multiplate and ROTEM, fish oil

	Before	After	*P*	95 % CI
Multiplate				
ADP (AUC)	73 ± 8.7	60 ± 5.9	0.003	−19.2 to −7.6
COL (AUC)	67 ± 6.7	64 ± 6.8	0.50	−15.2 to 8.8
ASPI(AUC)	77 ± 7.9	76 ± 11.8	0.86	−12.6 to 11.0
ROTEM				
EXTEM-CT (sec)	56 ± 10	61 ± 36	0.79	−48.4 to 59.2
EXTEM-CFT (sec)	103 ± 36	110 ± 31	0.81	−67.5 to 81.0
EXTEM-AA (degrees)	70 ± 6.5	68 ± 5.6	0.76	−15.1 to 11.9
EXTEM-MCF (mm)	62 ± 3.6	58 ± 4.3	0.23	−11.9 to 3.9
NATEM-CT (sec)	670 ± 68	728 ± 64	0.38	−102 to 216
NATEM-CFT (sec)	217 ± 32	284 ± 17	0.009	26.8 to 107
NATEM-AA (degrees)	52 ± 3.9	44 ± 2.3	0.009	−12.0 to −3.2
NATEM-MCF (mm)	51.7 ± 5.8	46.8 ± 3.9	0.18	−12.9 to 3.3

Results from Multiplate and ROTEM before and after peroral intake of 1260 mg Ω-3 polyunsaturated fatty acids (fish oil) for 7 days, mean ± SD. *CI* confidence interval. *ADP* adenosine diphosphate (6.5 μM). *AUC* area under curve. *COL* collagen (3.2 μg/ml). *ASPI* arachidonic acid (0.5 mM). *EXTEM* tissue factor triggered viscohemostatic test. *CT* clotting time. *CFT* clot formation time. *AA* α-angle. *MCF* maximal clot firmness. *NATEM* viscohemostatic test without coagulation activating reagents

### ROTEM

**Fig. 3:**
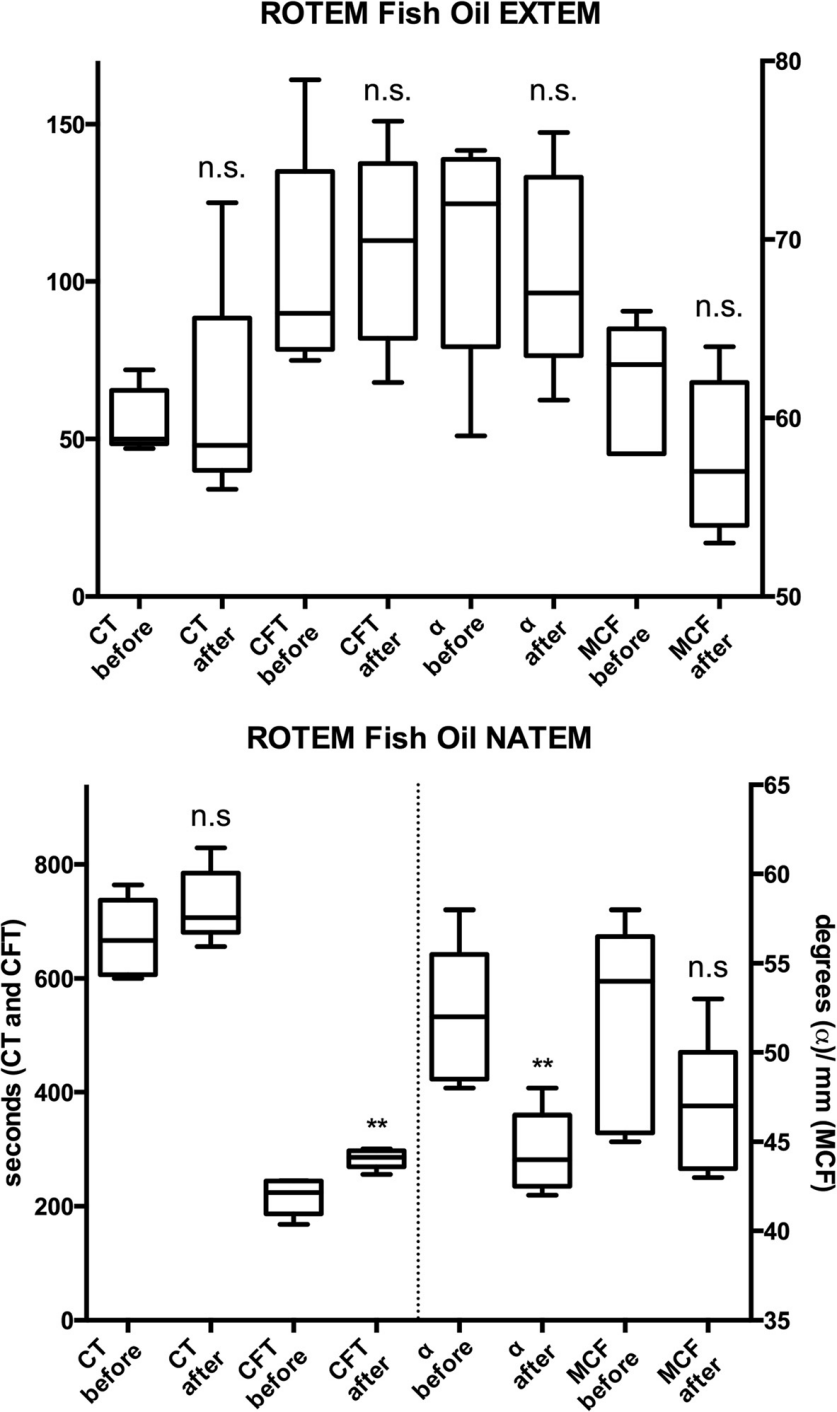
Results from ROTEM analysis of whole blood before and after 7 days of fish oil medication. Before and after results are compared using paired *t*-test. ** = *P* < 0.01. n.s. = non-significant

NATEM-CFT increased from 217 ± 32 s to 283 ± 20 s (*P* = 0.009, 95 % CI 26.8 to 107), and NATEM-AA decreased from 52 ± 3.9° to 44 ± 2.3° (*P* = 0.009, 95 % CI −12.0 to −3.2) after 7 days of fish oil intake (Fig. 3 and Table 2). Fish oil had no effect on the other NATEM or any EXTEM parameters.

There were no significant ROTEM changes with the other naturopathic medicines.

## Discussion

In this prospective single-centre pilot study, we have demonstrated that the intake of 1260 mg Ω-3 polyunsaturated fatty acids (fish oil) daily was the only one of 7 different naturopathic medications (also including echinacea, fish oil, ginkgo biloba, ginseng, St. John’s wort, valeriana and garlic) that showed significant changes in haemostatic point-of-care analyses. Multiplate ADP and ROTEM NATEM assays were weakened after 7 consecutive days with recommended peroral dosages of fish oil. These results suggest that it would be meaningful to perform a larger study with more healthy volunteers using ROTEM and Multiplate assays to measure the effect of some of the naturopathic medications included in this investigation.

The study was designed to reveal if any of these naturopathic medicines in recommended dosages could produce measurable effects on Multiplate and ROTEM aggregometry for the purposes of sample size calculation prior to a larger trial. These instruments are frequently used to detect defects in haemostasis in bleeding patients. Hodges et al. [2] reported that 22 to 37 % of patients and Adusumilli [1] reported that up to 57 % of patients undergoing elective surgery are using naturopathic medicines. The present study is warranted since use of naturopathic medicines is increasingly common and their use may be a factor to consider when treating bleeding patients, who are often monitored with the haemostatic instrument ROTEM (and sometimes Multiplate).

Fish oil has been associated with a 7 % lower risk of coronary heart disease mortality [22]. The exact mechanisms by which fish oil exerts such a protective function are still debated. The main mechanisms proposed include the lowering of plasma triglyceride levels, increased anti-inflammatory responses, plaque stabilisation and decreased platelet aggregation [23]. Furthermore, fish oil has been shown to reduce ADP-, adrenaline- and COL-induced platelet aggregation as well as P-selectin expression and to increase bleeding time [20]. Veljovic et al. [19] studied intravenous administration of 2100 mg Ω-3 polyunsaturated fatty acids 1 day and 4 h prior to coronary bypass surgery in a prospective randomized controlled trial. Compared to controls, Ω-3 polyunsaturated fatty acids significantly reduced postoperative platelet aggregation measured with Multiplate COL assay. Our results are therefore consistent with previous laboratory findings showing that fish oil reduces platelet aggregation and weakens coagulation. Even though fish oil has shown clear signs of decreasing platelet function and coagulation ability, the link to actual bleeding risk of these laboratory findings remains uncertain. Watson et al. [24] retrospectively reviewed the medical records of bleeding complications in patients treated with high-dose fish oil, clopidogrel and ASA compared with only clopidogrel and ASA and found no difference in bleeding incidence between the groups. In a recent review, Wachira et al. [25] described other protective mechanisms of fish oil even more important than platelet aggregation reduction and found no reason to stop fish oil intake before invasive procedures.

Point-of-care devices such as ROTEM and Multiplate have gained popularity during the last decade and are widely used in perioperative settings and in cardiology to monitor platelet-inhibiting drugs. Studies suggest that ROTEM is useful in guiding transfusion therapy [26] and diagnosing bleeding complications [27]. However both instruments have limitations and do not provide a complete picture of plasma coagulation and platelet function [28]. In the ROTEM instrument, coagulation is activated by direct addition of an activator, thus bypassing primary haemostasis and preventing detection of disorders of primary haemostasis as well as the presence of potent platelet inhibitory drugs. The Multiplate instrument detects defect pathways in platelets activated with specific agonists but may fail to identify other disorders, and it is also dependent on the number of platelets [29–31].

In the present study we showed decreased platelet aggregation and clot formation with Multiplate ADP and ROTEM NATEM assays after intake of fish oil. However, there were no significant effects on ROTEM or Multiplate aggregometry after ingestion of the other tested naturopathic medicines (garlic, ginseng, ginkgo biloba, echinacea, St. John’s wort and valeriana). These medicines have previously been shown to weaken coagulation and platelet function in other studies. Sirvastava et al. [32] performed an in vitro experiment in which an aqueous garlic extract was added to whole blood and found a dose-dependent inhibition of platelet aggregation with several different agonists using light transmission aggregometry. Chang et al. [33] added garlic extracts to blood from healthy volunteers and showed weakened platelet aggregation after adding collagen agonists in very low to very high concentrations. Kiesewetter et al. [34] also demonstrated reduced platelet aggregation in volunteers with cerebrovascular risk factors after daily intake of 800 mg of powdered garlic using a turbidometric platelet aggregation method. However, Scharbert et al. [35], using PFA-100 (agonists epinephrine, and collagen) and Multiplate (agonists arachidonic acid and collagen), failed to detect any platelet effects with very-low-dose dietary garlic in healthy volunteers. These results demonstrate, in contrast to our study, that high-dose garlic may decrease platelet aggregation. However, in many of these studies garlic extracts were added in vitro, which may not represent what happens in vivo, and one study [35] used very low doses of garlic. These circumstances may partly explain the different results in the previous studies and the present study.

Regarding the other naturopathic medications studied, ginseng has been proven to inhibit platelet activation [36]. In a study by Zhou et al., flow cytometry, Born aggregometry and Western blot were used to show that platelet function was inhibited via the mitogen-activated protein kinase pathway. Ginkgo biloba has been demonstrated to potentiate the ticlopidine inhibitory effect on ADP receptors in stroke patients [37]. In the perioperative setting, echinacea has been reported to be safe [38]. St. John’s wort exerts its effects by inhibiting serotonin, norepinephrine and dopamine reuptake by neurons. Like other serotonin reuptake inhibitors, it has the potential to depress platelet function. However, a direct effect on platelet function or coagulation has so far not been documented with St. John’s wort [4]. Valeriana is extensively used in Europe, is not always recommended to be discontinued before surgery, and has so far no documented increased bleeding risk [2, 4].

We were unable to demonstrate changes in platelet aggregation and coagulation after ingestion of naturopathic medicines other than fish oil. This suggests that Multiplate tests and ROTEM assays are not sensitive enough to assess the effects on the human coagulation system and platelets after medication with garlic, ginseng, ginkgo biloba, echinacea, St. John’s wort or valeriana, and that we need to consider using a more sensitive and specific method. It will probably be difficult to find a single method that can measure the effects on platelet function and coagulation of all the different medicines. Instead, we have to consider customizing methods by taking into account the mechanisms of each medicine.

There are few prospective studies on the impact of herb use on bleeding complications and surgical outcomes. Shalansky et al. [39] prospectively studied patient-assessed bleeding complications in patients using complementary and alternative medicine who were also treated with warfarin, and they found increased bleeding with coenzyme Q10 (odds ratio [OR] 3.69, 95 % CI 1.88 to 7.24) and ginger (OR 3.20, 95 % CI 2.42 to 4.24). Several cases of spontaneous intracranial bleeding, one case of spontaneous hyphema and one case of postoperative bleeding following laparoscopic cholecystectomy have been attributed to ginkgo use [4]. Ginsenosides inhibit platelet aggregation irreversibly, prolonging both the coagulation time of thrombin and activated partial thromboplastin. Lee et al. [40] demonstrated an increased risk of perioperative events for patients using 1 or more of 45 different traditional Chinese herbal medicines compared to patients not using these medicines. In contrast, Kepler et al. [41] found no increase in bleeding in patients using fish oil supplements prior to spinal decompression surgery. The study by Kepler et al. was well powered, making the negative result significant, but it is in disagreement with previous experimental studies demonstrating impaired platelet and coagulation function after fish oil ingestion. Further clinical studies are needed before any firm conclusions can be drawn. In anticipation of more such studies, experts have recommended avoiding herbs for at least 2 weeks prior to surgery [3]. This is a reasonable recommendation because stopping the naturopathic medications during this limited time is probably not harmful and may decrease the risk of perioperative complications.

We recognise the limitations of this study. The present study was a pilot study with low power and with the main aim of obtaining data that can provide a basis for sample size calculation before a larger trial. This means that even though we could not detect an effect on ROTEM and Multiplate assays of 6 out of 7 naturopathic medicines (echinacea, ginkgo biloba, ginseng, St. John’s wort, valeriana and garlic), there may still be bleeding risks related to these medicines, as shown by many others [5–9, 11, 12, 20] and also that the significant results must be interpreted with caution [42]. Furthermore, this study did not include platelet aggregation tests with agonists in different concentrations. This means that the question of whether naturopathic medications are able to inhibit platelet function at lower concentrations of agonists was not tested.

## Conclusion

We have demonstrated that the recommended peroral intake of 1260 mg Ω-3 polyunsaturated fatty acids (fish oil) daily was the only one of 7 different naturopathic medications (also including echinacea, fish oil, ginkgo biloba, ginseng, St. John’s wort, valeriana and garlic) that had a significant effect on Multiplate platelet aggregation and ROTEM viscoelastic clot formation. Fish oil caused decreased platelet aggregation after stimulation with standard-concentration ADP agonist (6.5 μM) in the Multiplate instrument and a prolonged clot formation time in the NATEM assay in the ROTEM instrument. These results must be interpreted with some caution due to low statistical power and should be confirmed in larger trials.
